# Neonatal Milk Fat Globule Membrane Supplementation During Breastfeeding Ameliorates the Deleterious Effects of Maternal High-Fat Diet on Metabolism and Modulates Gut Microbiota in Adult Mice Offspring in a Sex-Specific Way

**DOI:** 10.3389/fcimb.2021.621957

**Published:** 2021-03-19

**Authors:** Lin Ye, Qianren Zhang, Fengzhi Xin, Baige Cao, Linxi Qian, Yan Dong

**Affiliations:** ^1^ Xinhua Hospital, Shanghai Institute for Pediatric Research, Shanghai Jiao Tong University, School of Medicine, Shanghai, China; ^2^ Shanghai Key Laboratory of Pediatric Gastroenterology and Nutrition, Xinhua Hospital, Shanghai Jiao Tong University, School of Medicine, Shanghai, China; ^3^ Department of Gastroenterology, Xinhua Hospital, Shanghai Jiao Tong University, School of Medicine, Shanghai, China; ^4^ Department of Endocrinology, Xinhua Hospital, Shanghai Jiao Tong University, School of Medicine, Shanghai, China

**Keywords:** gut microbiota, milk fat globule membrane, high-fat diet, sex dimorphism, metabolic disorder

## Abstract

Exposure to adverse events in early life increases the risk of chronic metabolic disease in adulthood. The objective of this study was to determine the significance of milk fat globule membrane (MFGM)-mediated alterations in the gut microbiome to the metabolic health of offspring in the long-term. Female C57BL/6 mice were fed either a high-fat diet (HFD) or a control diet for 3 weeks before pregnancy and throughout pregnancy and lactation. During lactation, pups from the HFD group were breast-fed with or without 1,000 mg/kg BW/day MFGM supplementation (HFD and HFD-MS group, respectively). After weaning, the offspring in each group were divided into male and female subgroups. The weaned mice were then shifted to a control diet for 8 weeks. At the eleventh week, stool samples were collected for 16S rRNA gene sequencing. Serum biochemical parameters were analyzed, and intraperitoneal glucose and insulin tolerance tests were performed. Neonatal supplementation with MFGM ameliorated metabolic disorder and improved glucose tolerance in offspring exposed to maternal HFD in a sex-specific manner. Furthermore, maternal HFD induced gut microbiota perturbation in offspring in adulthood. Neonatal MFGM supplementation significantly enriched *g-Parabacteroides*, *g-Bifidobacterium*, *g-Faecalibaculum*, and *g-Lactobacillus* in male offspring exposed to maternal HFD, while significantly enriched *g-Parabacteroides* and *g-Alistipes* in female offspring exposed to maternal HFD. These bacteria may be associated with the favorable changes in metabolism that occur in adulthood. Sex differences in the changes of metagenomic pathways related to oxidative phosphorylation, citrate cycle, electron transfer carries, and ubiquinone biosynthesis were also observed in the offspring. Maternal HFD has an adverse effect on the metabolism of offspring in later life. Neonatal MFGM supplementation could modulate the structure of gut microbiota communities and may have long-term protective effects on lipid and glucose metabolism, but these effects are sex dimorphic.

## Introduction

Childhood obesity remains a major public health problem and is associated with metabolic disorders later in life, including insulin resistance, cardiovascular disease (CVD), and non-alcoholic fatty liver disease (NAFLD). Clinical and experimental studies have linked maternal obesogenic environments to the development of obesity and related metabolic disorders in offspring (Keleher et al., 2018). The prenatal and infancy stages are considered particularly critical time windows in determining the individual risk of developing obesity in later life. The theory of the developmental origins of health and disease (DOHaD) proposed by David Barker states that fetal or infant exposure to adverse factors, such as poor nutrition and adverse intrauterine environment, could increase the risk of chronic diseases, including obesity, diabetes, and CVD, in adulthood (Barker and Osmond, 1986; Barker, 2007). Both maternal overnutrition and malnutrition are considered risk factors for offspring obesity. Maternal obesity or excessive weight gain during pregnancy has been associated with adiposity in children. Maternal gestational hyperglycemia and diabetes can affect fetal glucose control and increase the risk of adolescent adiposity and metabolic disorders (Arenz et al., 2004). The impact of maternal nutrition on offspring metabolic health has also been studied extensively in animal models. A large number of animal studies have shown that a maternal high-fat diet (HFD) during gestation and lactation could “program” offspring toward metabolic diseases and have lasting effects on their long-term health (Ribaroff et al., 2017; Mann et al., 2018).

Although many factors are involved in the transmission of metabolic diseases from mother to child, such as genetic, epigenetic factors, and environmental influence, early life microbial symbiosis has been considered a crucial factor for the long-term metabolic health issues (Hoffman et al., 2017; Milani et al., 2017). The route of mother-to-fetus microbial transmission is still controversial, with opinions ranging from *in-utero* to postpartum colonization. Numerous studies have demonstrated that gut microbiota dysbiosis caused by maternal transmission could result in obesity and other metabolic diseases (Zhou and Xiao, 2018), which may be passed to subsequent generations. Maternal obesity was found to modulate the gut microbiota of offspring and affect their nutrient acquisition and energy homeostasis, indicating that an obesogenic trait is transmitted from mother to child. A follow-up study showed that obese or overweight pregnant women and women with excessive gestational weight gain had a more proinflammatory gut microbiome than their lean counterparts (Collado et al., 2008). In a murine model, a previous study showed that dams fed HFD throughout gestation and lactation produced offspring with gut microbiota dysbiosis and metabolic disorder, which persisted into adulthood (Guo et al., 2018). Gut microbiota dysbiosis in early life due to maternal HFD exposure may have consequences on metabolic health later in life. Therefore, reshaping the infant gut microbiome using dietary agents is a promising strategy to break this cross-generational transmission of obesity-related outcomes.

Breast milk contains many biologically active ingredients that are important for the development of infants, such as oligosaccharides, probiotics, immunoglobulins, and growth factors. Breast milk is also a source of live bacteria and contains energy sources for colonizing bacteria (Milani et al., 2017). Milk fat is an important component of milk, usually found in the form of milk fat globule (MFG). During the process of milk fat secretion, MFG is surrounded by a triple phospholipid and cholesterol layer, known as the milk fat globule membrane (MFGM). MFGM envelops the triacylglycerol core of the MFG, playing an emulsifying and stabilizing role in milk and preventing MFG aggregation and degradation (Jukkola and Rojas, 2017).

The MFGM in breast milk is composed of several bioactive ingredients, including phospholipids, glycoproteins, and enzymes. MFGM is the sole source of phospholipids in breast milk and is primarily comprised of sphingomyelins, phosphatidylcholines, and phosphatidylethanolamines (Timby et al., 2017). It has been demonstrated that the dietary administration of phospholipids or sphingomyelins may prevent body weight gain, improve lipid metabolism, and alter gut microbiota composition in HFD-fed adult mice (Norris et al., 2016; Milard et al., 2019). Furthermore, Li et al. found that the supplementation of phospholipid-enriched MFGM to HFD dams during pregnancy and lactation promoted brown/beige adipogenesis and provided protection against maternal HFD-induced adiposity in offspring. However, MFGM is a milk-derived nutrient supply. A better mimic of its physiological role would be to supply MFGM to pups during suckling and observe its protective effects against maternal-transmitted metabolic disorders. Although a previous study showed a direct connection between MFGM supplementation and the neonatal gut microbiome (Bhinder et al., 2017), no study has investigated whether neonatal MFGM consumption has long-term effects on the composition of the adult gut microbiota.

In a previous study, we found that MFGM supplementation during suckling exerted long-term protective effects against a predisposition to maternal HFD-induced hepatic steatosis in offspring. However, the exact mechanism is not well understood. Further research is needed to define the significance of neonatal MFGM supplementation on the long-term metabolic health of offspring. Therefore, we continued to explore the influence of neonatal MFGM supplementation on lipid and glucose metabolism in adult offspring mice. In addition, we investigated the role of MFGM supplementation on modulating adult gut microbiota and the correlation between metabolic outcomes and altered bacteria.

## Materials and Methods

### Animals and Diets

All animal experiments were approved by the Institutional Review Board and the Animal Care and Use Committee of Shanghai Xinhua Hospital. Five-week-old virgin female C57BL/6 mice were purchased from SLAC Laboratory Animal Co. (China). All mice were housed in ventilated cages with a 12:12 h light-dark cycle at a constant temperature (21–23°C) and with free access to food and water. After acclimatizing for 1 week, the mice were randomly divided into two groups. The mice in the different groups were fed a control diet (16.7% kcal from fat, 64% kcal from carbohydrate, and 19.3% kcal from protein; LAD3001G, TROPHIC Animal Feed High-Tech Co. LTD, China) or a high-fat diet (60% kcal from fat, 20.6% kcal from carbohydrate, and 19.4% kcal from protein; TP23300, TROPHIC Animal Feed High-Tech Co. LTD, China) for 3 weeks before mating. Details of dietary differences are shown in **Supplementary Table 1**. Female mice were subsequently mated with 10-week-old male C57BL/6 mice, fed a control diet, at a ratio of two females to one male for approximately 1 week. During pregnancy and lactation, the mice in each group were fed their corresponding diet. Mating success was confirmed by checking for vaginal plug formation. Upon pregnancy, the breeding pairs were separated and the female mice were housed individually until the end of lactation stage. The first appearance of a litter from primiparous dams was recorded as postnatal day (PD) 1. Offspring were not handled until postnatal day 3 (PD3) for fear of being bitten by dams. At PD3, each litter was adjusted to six pups to ensure similar milk intake.

**Scheme 1:**
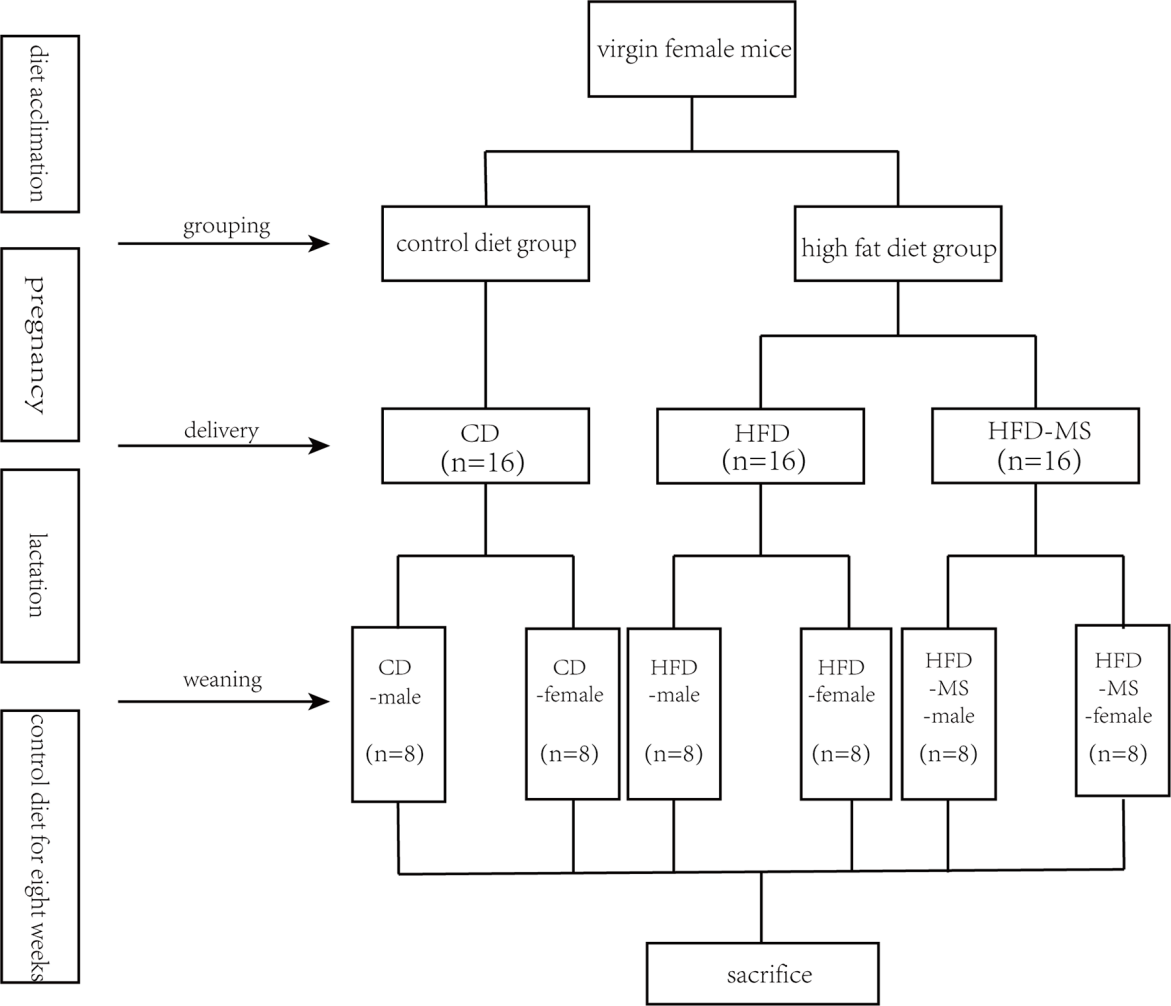
Experimental groups. Five-week-old C57BL/6J mouse dams were fed CD or HFD 3 weeks before mating and throughout pregnancy and lactation. During suckling, offspring from HFD dams were supplemented without or with 1,000 mg/kg body weight/day MFGM. After weaning at 3 weeks, all offspring were fed CD for 8 weeks. Male and female offspring were sacrificed and assessed at 11 weeks of age. CD, control diet; HFD, high-fat diet; MS, milk fat globule membrane supplementation.

Pups exposed to a maternal HFD were randomly placed into two groups: breast-fed without MFGM supplementation (HFD group) and breast-fed with 1,000 mg/kg BW/day MFGM supplementation (HFD-MS group). The MFGM concentration used in this study was calculated based on the amount of phospholipids in rodent breast milk. This concentration was close to the dosage reported by Bhinder et al. (2017). The bovine MFGM was prepared using the protocol described in our previous study (Zhang et al., 2019). From PD3 to PD21, a 100 mg/ml suspension of MFGM dispersed in ddH_2_O was administered daily in the HFD-MS group by oral gavage, while the offspring from the control diet-fed dams (CD group) and HFD group received the same volume of vehicle (ddH_2_O) daily. After weaning, the offspring were divided based on sex in all groups and the number of offspring in each group is eight. A male suffix was added after the original group names in the male groups, while the female suffix was added to distinguish them from male groups. All mice consumed a control diet for 8 weeks until they were sacrificed at 11 weeks of age. A schematic diagram of the experimental design is shown in **Scheme 1**.

On the day before the end of the experiment, fresh stool samples were collected for gut microbiota analysis and then frozen in liquid nitrogen. At the end of the experiment, the offspring were anesthetized with 1% pentobarbital and euthanized *via* cervical dislocation. Blood samples were obtained from the retro-orbital sinus after fasting overnight, and then incubated at room temperature for 2 h. Serum was collected by centrifugation at 5,000 rpm for 30 min at 4°C and stored at ‒80°C.

### Intraperitoneal Glucose Tolerance Test (IPGTT) and Intraperitoneal Insulin Tolerance Test (IPITT)

IPGTT and IPITT were performed either 1 or 2 weeks before euthanasia, respectively. For IPGTT, 14–16 h fasted mice were administered 50% glucose solution (2 g/kg BW) by intraperitoneal injection. Blood was collected from the tail vein at 0, 15, 30, 60, and 120 min after administration, and the blood glucose level was measured with a glucometer (Bayer Healthcare LLC, Germany). For IPITT, mice fasted for 6 h were injected intraperitoneally with insulin (Sigma-Aldrich, USA) at a dose of 0.75 U/kg BW. The time points of blood collection and the methods of estimation were identical to those of the IPGTT. The glucose response was evaluated using the area under the curve (AUC).

### Serum Biochemical Analysis

Total serum cholesterol (TC), triglyceride (TG), alanine aminotransferase (ALT), aspartate aminotransferase (AST), fasting blood glucose (FBG), low-density lipoprotein cholesteryl ester (LDL-C), and high-density lipoprotein cholesteryl ester (HDL-C) were estimated using an automatic biochemistry analyzer (Sysmex CHEMIX-180, Japan). The fasting insulin levels were determined using a commercial insulin enzyme-linked immunosorbent assay kit (Crystalchem, USA). All procedures were conducted according to the manufacturer’s instructions.

### Microbial DNA Extraction and Gene Sequencing

The stool samples of 11-week-old mice in all groups were sent to the Shanghai OE Biotech. Co., Ltd. (Shanghai, China) for 16S rRNA gene sequencing. Bacterial DNA was extracted from the fecal contents according to the manufacturer’s instructions (DNeasy PowerSoil Kit; Qiagen, Germany). The quality and quantity of DNA were verified by NanoDrop and agarose gel electrophoresis. The extracted DNA was diluted to a concentration of 1 ng/μl and stored at ‒20°C until further processing. The diluted DNA was used as a template for the PCR amplification of bacterial 16S rRNA genes with the barcoded primers and Takara Ex Taq (Takara, Japan). For bacterial diversity analysis, the V3-V4 variable regions of the 16S rRNA genes were amplified using the universal primers 343F-5’-TACGGRAGGCAGCAG-3’, and 798R-5’- AGGGTATCTAATCCT-3’. The amplicon quality was visualized using gel electrophoresis, purified with Agencourt AMPure XP beads (Beckman Coulter, USA), and amplified for another round of PCR. After purifying again with AMPure XP beads, the final amplicon was quantified using a Qubit dsDNA assay kit (Thermo Fisher Scientific, USA). Equal amounts of purified amplicon were pooled for sequencing using the Illumina MiSeq platform.

Raw sequencing data were obtained in FASTQ format. Paired-end reads were then preprocessed using Trimmomatic (version 0.35) software to detect and remove ambiguous bases (N) (Bolger et al., 2014). Low-quality sequences with an average quality score below 20 were also removed using a sliding window trimming approach. After trimming, the paired-end reads were assembled using FLASH (version 1.2.11) software (Reyon et al., 2012). The parameters of assembly were as follows: minimal overlapping, 10 bp; maximum overlapping, 200 bp; maximum mismatch rate, 20%. The sequences were subjected to further denoising as follows: reads with ambiguous, homologous sequences, or sequences below 200 bp were abandoned. Reads with 75% of bases above Q20 were retained. Thereafter, any reads with chimera were detected and removed. These two steps were performed using QIIME (version 1.8.0) software (Caporaso et al., 2010). Clean reads were subjected to primer sequence removal and clustering to generate operational taxonomic units (OTUs) using Vsearch (version 2.4.2) software with a 97% similarity cut-off (Rognes et al., 2016). The representative read of each OTU was selected using QIIME. All representative reads were annotated and blasted against the Silva database (version 123) using the RDP classifier (confidence threshold, 70%) (Wang et al., 2007).

### Bioinformatic Analysis

To clarify the similarities between the composition of fecal microbiota among all the experimental groups in adult male and female offspring, the estimators of α-diversity, including community richness (Chao 1) and diversity (Simpson and Shannon index), were calculated based on rarefied OTU tables. Principal coordinates analysis (PCoA) and unweighted pair group method with arithmetic mean (UPGMA) on unweighted UniFrac distance matrix were displayed as the β-diversity. The results of linear discriminant analysis coupled with effect size measurements (LEfSe) revealed different gut microbiota from the phylum level down to the genus level among the experimental groups. Phylogenetic investigation of communities by reconstruction of unobserved states (PICRUST) was used to predict the 16S rRNA-based high-throughput sequencing data for functional features from the phylogenetic information. The predicted functional composition profiles were then collapsed into level 2 and 3 of KEGG (Kyoto Encyclopedia of Genes and Genomes) database pathways. The correlations between the significantly different bacteria at the genus level and the serum parameters among the mice offspring were determined by RDA (redundancy analysis) and CCA (canonical correspondence analysis) and the related heatmap figures.

### Statistical Analysis

The data are expressed as the mean ± standard error of the mean (SEM). One-way analysis of variance (ANOVA) was performed to compare the biochemical parameters, body weight, AUC, and α-diversity indices among multiple groups. Two-way ANOVA was performed to compare the blood glucose levels of IPGTT and IPITT at different time points among multiple groups. Tukey’s test was used to assess the statistical significance between groups following ANOVA tests. Differences in the relative abundance of gut microbiota were analyzed using the Kruskal–Wallis test with *post-hoc* analyses. Correlation analyses between the relative abundance of bacterial taxa at the genus level and metabolic parameters were performed using the Spearman correlation coefficient test. P-value <0.05 was considered statistically significant. Prism version 7.0 (GraphPad Software Inc., San Diego, CA, USA) was used for statistical analysis.

## Results

### Neonatal Supplementation of MFGM Does Not Alter Body Weight of Adult Male and Female Offspring Exposed to Maternal HFD

**Figure 1:**
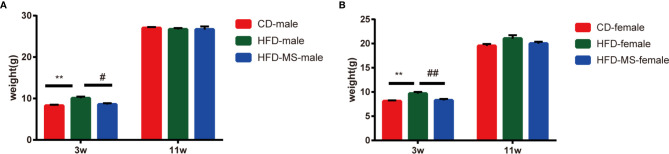
Body weight of adult male and female offspring. **(A)** Body weight of male mice at 3 or 11 weeks. **(B)** Body weight of female mice at 3 or 11 weeks. Data are expressed as the mean ± SEM. N = 6–8/group. **p < 0.01 *vs* the CD group; ^#^p < 0.05 and ^##^p < 0.01 *vs* the HFD group. CD, control diet; HFD, high-fat diet; MS, milk fat globule membrane supplementation.

Changes in the body weight of male and female offspring were measured weekly from the weaning day (PD21) until 11 weeks of age. As shown in **Figure 1**, the body weight in the HFD group was significantly higher compared to the CD group in both male (HFD-male: 10.15 ± 0.42 g *vs* CD-male: 8.42 ± 0.19 g; P < 0.01) and female (HFD-female: 9.67 ± 0.35 g *vs* CD-female: 8.09 ± 0.17 g; P < 0.01) offspring at weaning. By contrast, MFGM supplementation reduced the body weight of male (HFD-male: 10.15 ± 0.42 g *vs* HFD-MS-male: 8.78 ± 0.31 g; P < 0.05) and female (HFD-female: 9.67 ± 0.35 g *vs* HFD-MS-female: 8.25 ± 0.30 g; P < 0.01) offspring in the HFD-MS group compared to the HFD group at weaning. Further comparison was conducted on the body weight of adult offspring at 11 weeks of age, which showed no significant difference among the CD, HFD, and HFD-MS groups in either male or female offspring (**Figures 1A, B**).

### Neonatal Supplementation of MFGM Ameliorates Metabolic Disorders in Adult Male and Female Offspring Exposed to Maternal HFD

Although no significant difference was observed in body weight at 11 weeks among the three groups in both male and female offspring, the serum biochemical parameters related to lipid and glucose metabolism were examined in the adult mice offspring. The serum parameters of adult male and female offspring are summarized in **Table 1**. In the adult male offspring, there were no significant changes in serum TC and LDL-C in the HFD group compared to the CD group. However, the TG content in the HFD group was much higher than that in the CD group (P < 0.01). MFGM supplementation during suckling attenuated the increase in serum TG caused by maternal HFD (P < 0.01). The HDL-C levels in the HFD group were significantly lower than those in the CD group (P < 0.05), whereas MFGM supplementation slightly increased the HDL-C levels (P = 0.06). Glucose metabolism showed that the concentration of FBG in the HFD group was significantly higher than that in the CD group (P < 0.01), and MFGM supplementation during suckling normalized FBG in the HFD-MS group (P < 0.01). Exposure to HFD is known to increase the accumulation of lipid droplets in the liver, and subsequent hepatic injury. Therefore, we measured the levels of ALT and AST in the serum of all groups. As a result, the serum ALT and AST levels were found to be significantly higher in the male offspring from the HFD group than in those from the CD group (P < 0.01), whereas neonatal MFGM supplementation resulted in significant improvements (P < 0.01).

**Table 1:** Serum biochemistry parameter.

Biochemistry parameter	CD (n = 6–8)	HFD (n = 6–8)	HFD-MS (n = 6–8)
**Male**			
TG (mmol/L)	0.58 ± 0.05	0.96 ± 0.10^**^	0.60 ± 0.04^##^
TC (mmol/L)	2.32 ± 0.19	2.71 ± 0.08	2.70 ± 0.06
HDL-C (mmol/L)	2.33 ± 0.07	1.90 ± 0.09^*^	2.27 ± 0.15
LDL-C (mmol/L)	0.32 ± 0.01	0.35 ± 0.02	0.28 ± 0.02^#^
AST (U/L)	116.13 ± 9.54	203.38 ± 21.61^**^	109.50 ± 6.42^##^
ALT (U/L)	22.63 ± 2.15	62.38 ± 12.71^**^	23.38 ± 2.15^##^
FBG (mmol/L)	2.14 ± 0.26	5.00 ± 0.62^**^	2.85 ± 0.34^##^
INSULIN (mU/L)	9.94 ± 1.07	9.37 ± 1.38	8.95 ± 1.06
**Female**			
TG (mmol/L)	0.51 ± 0.04	0.65 ± 0.03	0.69 ± 0.09
TC (mmol/L)	2.00 ± 0.09	1.98 ± 0.06	2.06 ± 0.07
HDL-C (mmol/L)	1.55 ± 0.06	1.15 ± 0.04^**^	1.50 ± 0.04^##^
LDL-C (mmol/L)	0.33 ± 0.04	0.45 ± 0.11	0.34 ± 0.03
AST (U/L)	117.6 ± 3.91	142.00 ± 2.73^**^	115.40 ± 4.36^##^
ALT (U/L)	23.63 ± 0.92	24.63 ± 0.96	25.50 ± 1.04
FBG (mmol/L)	2.73 ± 0.35	3.72 ± 0.53	2.06 ± 0.20^#^
INSULIN (mU/L)	7.71 ± 0.92	9.27 ± 0.86	7.98 ± 1.08

Data are expressed as means ± SEM (n = 6–8/group). ∗p < 0.05, ∗∗p < 0.01 vs the CD group; ^#^p < 0.05, ^##^p < 0.01 vs the HFD group. CD, control diet; HFD, high-fat diet; HFD-MS, high-fat diet with milk lipid globule membrane supplementation.

In the adult female offspring, maternal HFD exposure did not alter the levels of TG, TC, or LDL-C, but decreased the level of HDL-C (P < 0.01). Change in the HDL-C levels caused by maternal HFD were recovered by neonatal supplementation with MFGM (P < 0.01). Additionally, maternal HFD was found to increase the level of AST but not ALT in the HFD group (P < 0.01), and MFGM supplementation alleviated the injury (P < 0.01). Unlike male offspring, there was no significant difference in fasting blood glucose and insulin levels between the HFD and CD groups. Despite this, neonatal MFGM supplementation still reduced the FBG in female offspring (P < 0.05).

### Neonatal Supplementation of MFGM Improves Glucose Tolerance and Insulin Sensitivity in Adult Male Offspring Exposed to Maternal HFD

**Figure 2:**
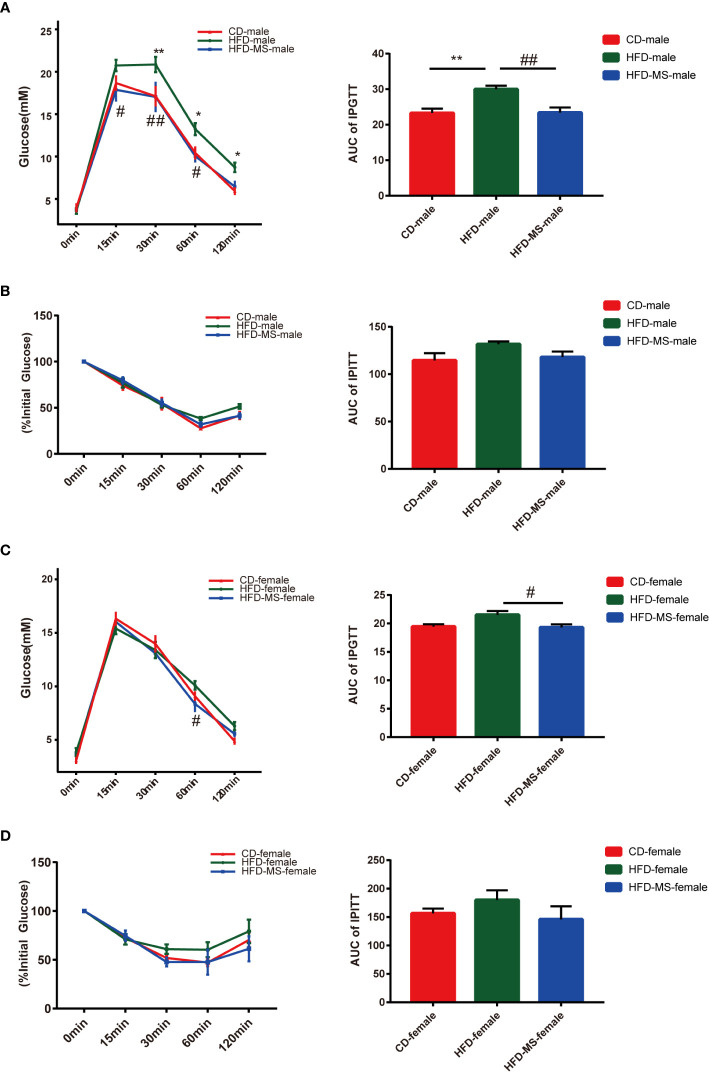
Glucose metabolism of adult male and female offspring. **(A)** IPGTT and AUC of adult male mice. **(B)** IPITT and AUC of adult male mice. **(C)** IPGTT and AUC of adult female mice. **(D)** IPITT and AUC of adult female mice. Data are expressed as the mean ± SEM. N = 5–7/group. *p < 0.05 and **p < 0.01 *vs* the CD group; ^#^p < 0.05 and ^##^p < 0.01 *vs* the HFD group. IPGTT, intraperitoneal glucose tolerance test; IPITT, intraperitoneal insulin tolerance test; AUC, area under the curve; CD, control diet; HFD, high-fat diet; MS, milk fat globule membrane supplementation.

Since a higher level of FBG was observed in the HFD group compared to the CD group, IPGTT and IPITT were performed to determine the effect of MFGM on glucose metabolism in offspring mice. As shown in **Figure 2A**, according to the IPGTT, the blood glucose levels were higher at 30 min (P < 0.01), 60 min (P < 0.05), and 120 min (P < 0.05), and the AUC was significantly larger in adult male offspring from the HFD group than in those from the CD group (P < 0.01). In the HFD-MS group, the glucose tolerance impaired by maternal HFD was found to be improved by MFGM supplementation (P < 0.01). According to the IPITT (**Figure 2B**), there was no significant difference at different times after administering insulin among groups in the adult male offspring. Correspondingly, there was also no significant difference in the AUC of IPITT.

According to IPGTT and IPITT, the glucose tolerance and insulin sensitivity in adult female offspring were resistant to maternal HFD exposure (**Figures 2C, D**). Notwithstanding the lack of differences in the tolerance and sensitivity tests between the HFD and CD groups, the neonatal supplementation of MFGM was found to reduce the AUC of IPGTT curve (P < 0.05).

### Neonatal Supplementation of MFGM Alters Fecal Microbiota in Adult Male and Female Offspring Exposed to Maternal HFD

**Figure 3:**
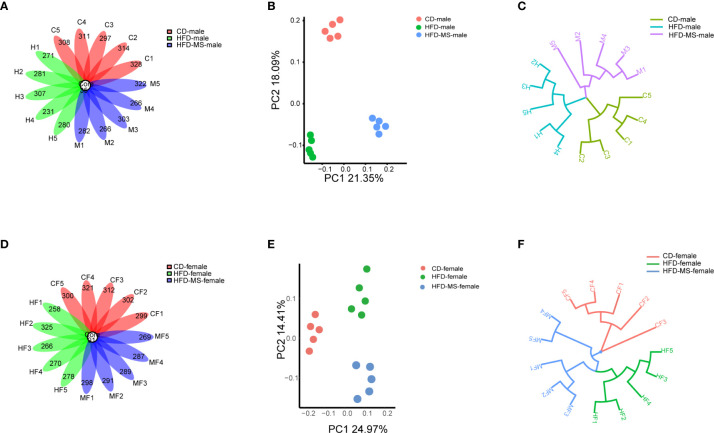
Different microbial diversity indices in different groups. **(A)** Flower plots of OTUs in adult male mice. **(B)** β-diversity analysis of microbial communities using PCoA based on unweighted Unifrac distance in the adult male. **(C)** β-diversity analysis of microbial communities using UPGMA based on unweighted Unifrac distance in the adult male mice. **(D)** Flower plots of OTUs in adult female mice. **(E)** β-diversity analysis of microbial communities using PCoA based on unweighted Unifrac distance in the adult female offspring. **(F)** β-diversity analysis of microbial communities using UPGMA based on unweighted Unifrac distance in the adult female mice. N = 5/group. CD, control diet; HFD, high-fat diet; MS, milk fat globule membrane supplementation; OTU, operational taxonomic unit; PCoA, principal coordinate analysis; UPGMA, unweighted pair group method with arithmetic mean.

To explore the mechanism by which maternal HFD induced offspring metabolic disorders and MFGM supplementation during suckling prevented metabolic disorders, we performed 16s rRNA sequencing of the fecal flora of adult male and female offspring. The OTUs were annotated, and the shared and unique OTUs among groups were indicated by flora plots (**Figures 3A, D**). As a result, the α-diversity indices Chao 1, Simpson, and Shannon were found to not be significantly different among the three groups in either male or female offspring (**Supplementary Figure 1**). β-diversity analysis of the fecal communities using PCoA (**Figures 3B, E**) and UPGMA (**Figures 3C, F**) based on unweighted Unifrac distance revealed that the overall microbial compositions of the CD, HFD, and HFD-MS groups separated from each other in the male and female offspring.

**Figure 4:**
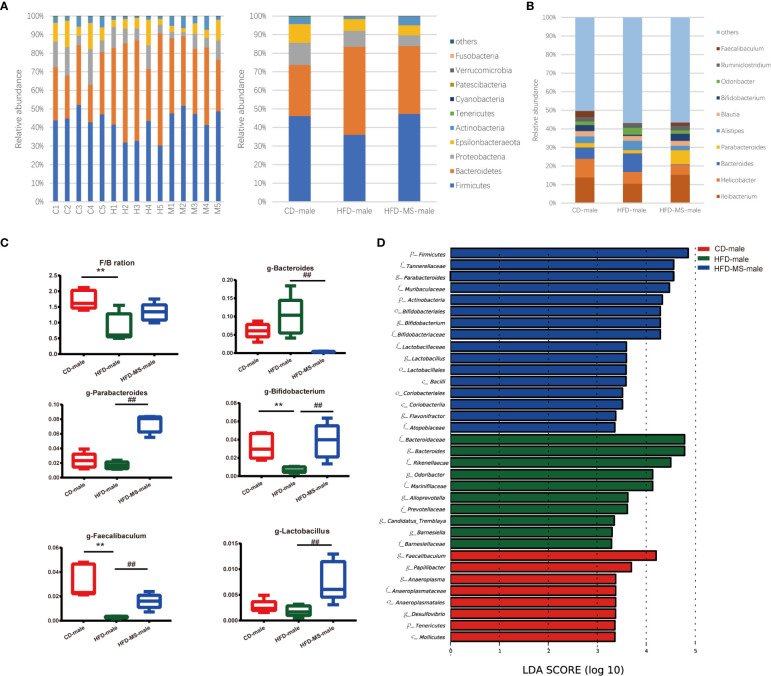
Changes in the gut microbiota of adult male offspring. **(A)** Relative abundance of bacterial taxa at the phylum level in each male mouse and among three groups. **(B)** Relative abundance of bacterial taxa at the genus level among three groups. **(C)** Significantly different genera and F/B ratio. **(D)** LEfSe analysis of the different gut microbiota from the phylum level to the genus level among three groups. N = 5/group. **p < 0.01 *vs* the CD group; and ##p < 0.01 *vs* the HFD group. CD, control diet; HFD, high-fat diet; MS, milk fat globule membrane supplementation; F/B, *Firmicutes*/*Bacteroidetes*; LEfSe, linear discriminant analysis (LDA) coupled with effect size measurements.

**Figure 5:**
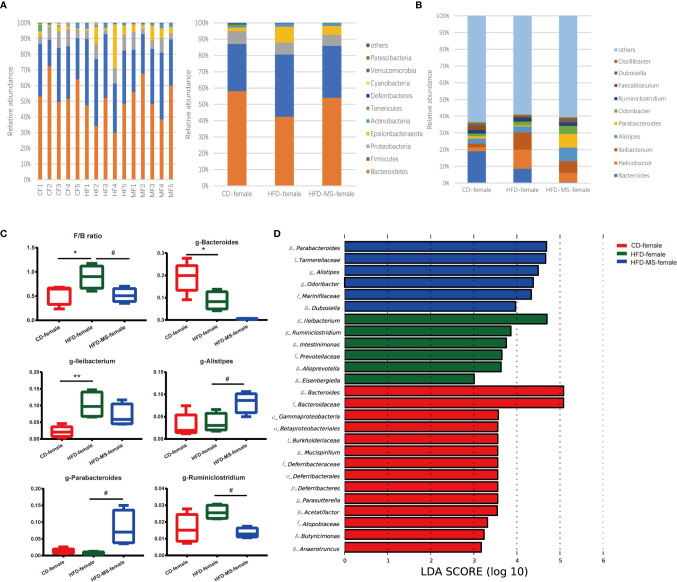
Changes in the gut microbiota of adult female offspring. **(A)** Relative abundance of bacterial taxa at the phylum level in each female mouse and among three groups. **(B)** Relative abundance of bacterial taxa at the genus level among three groups. **(C)** Significantly different genera and F/B ratio. **(D)** LEfSe analysis of the different gut microbiota from the phylum level to the genus level among three groups. N = 5/group. *p < 0.05 and **p < 0.01 *vs* the CD group; ^#^p < 0.05 *vs* the HFD group. CD, control diet; HFD, high-fat diet; MS, milk fat globule membrane supplementation; F/B, *Firmicutes*/*Bacteroidetes*; LEfSe, linear discriminant analysis (LDA) coupled with effect size measurements.

We compared the relative abundance of the bacterial communities among the groups by measuring the number of observed OTUs. At the phylum level, *p-Bacteroidetes* and *p-Firmicutes* were found to be the two most predominant taxa in both male and female offspring (**Figures 4A**, **5A**). In the adult male offspring, we found that maternal HFD reduced the *Firmicutes*/*Bacteroidetes* (F/B) ratio relative to the CD group (P < 0.01) (**Figure 4C**). **Figure 4B** shows the proportions of the top 10 most abundant genera in the three groups. The relative abundance of *Bifidobacterium* and *Faecalibaculum* was significantly decreased in the HFD group compared to the CD group (P < 0.01), while MFGM supplementation during suckling reversed these altered genera (P < 0.01) (**Figure 4C**). The relative abundance of *g-Bacteroides* was distinctly lower and that of *g-Parabacteroides* and *g-Lactobacillus* was distinctly higher in the male offspring from the HFD-MS group than in those from the HFD group (P < 0.01), notwithstanding no differences in these two genera between the CD and HFD groups (**Figure 4C**). LEfSe analysis demonstrated significantly different microbiota among the groups of male offspring at the phylum, class, order, family, and genus levels (**Figure 4D**).

In the adult female offspring, maternal HFD increased the F/B ratio relative to the CD group (P < 0.05). However, neonatal MFGM supplementation shifted the F/B ratio in the HFD-MS group close to that in the CD group (P < 0.05) (**Figure 5C**). The proportions of the top 10 most abundant genera in the three groups are shown in **Figure 5B**. We found that the relative abundance of *g-Bacteroides* decreased (P < 0.05) and that of *g-Ileibacterium* increased (P < 0.01) in the HFD group compared with the CD group (**Figure 5C**). Furthermore, when compared with the HFD group, the relative abundance of *g-Alistipes* and *g-Parabacteroides* increased (P < 0.05), whereas that of *g-Ruminiclostridium* decreased in the HFD-MS group (P < 0.05) (**Figure 5C**). LEfSe analysis demonstrated significantly different microbiota among the groups of female offspring at the phylum, class, order, family, and genus levels (**Figure 5D**).

### Functional Prediction of Gut Microbiota Among Groups in Adult Male and Female Offspring

**Figure 6:**
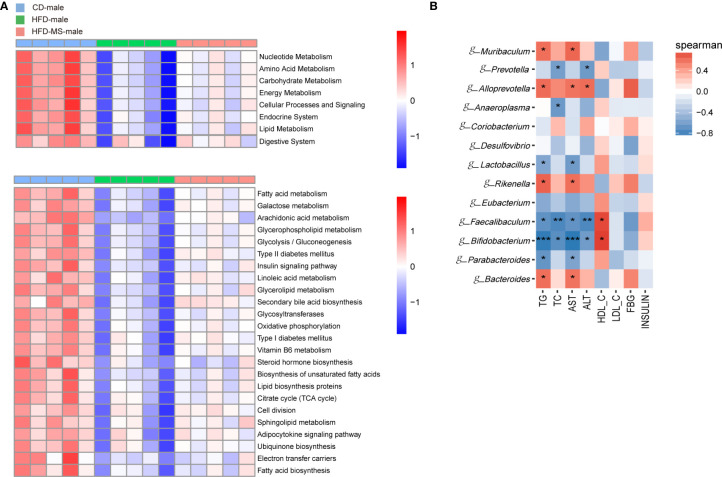
Microbial functional features and correlation analysis between the altered genera with the serum parameters in adult male offspring. **(A)** KEGG (Kyoto Encyclopedia of Genes and Genomes) database pathways were used to predict the microbial functional features at levels 2 and 3 among three groups in the adult male offspring. **(B)** Heatmap of Spearman correlation analysis between the significantly different germs at the genus level with serum biochemical parameters among different groups in the adult male offspring. N = 5/group. *p < 0.05, **p < 0.01, and ***p < 0.001 indicate significant Spearman correlation. CD, control diet; HFD, high-fat diet; MS, milk fat globule membrane supplementation.

In the enrichment analysis of KEGG-derived pathways based on high-throughput sequencing data, significant differences were found among the three groups in both male and female adult offspring. In the male offspring, we found a lower abundance of pathways related to the metabolism of nutrients, including carbohydrate, lipid, amino acid and nucleotide, and some systems such as endocrine and digestive system in the HFD group compared with the CD group. Neonatal MFGM supplementation increased the abundance of these pathways to various degrees in the HFD-MS group (**Figure 6A**). Furthermore, maternal HFD decreased the abundance of some functional pathways in the HFD group in the level 3 category relative to the CD group. These functional pathways are mainly involved in biological processes related to lipid and energy metabolism, such as unsaturated fatty acid biosynthesis, linoleic acid metabolism, sphingolipid metabolism, glycosphingolipid metabolism, adipocytokine signaling transduction, glycolysis/gluconeogenesis, oxidative phosphorylation, citrate cycle, and electron transfer carries. However, the attenuated richness of these pathways in the HFD group was recovered in the HFD-MS group (**Figure 6A**).

**Figure 7:**
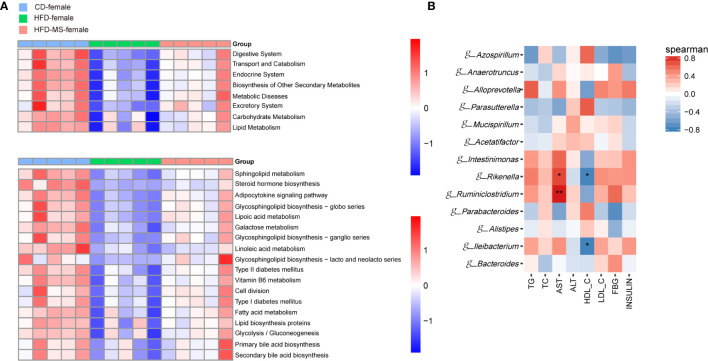
Microbial functional features and correlation analysis between the altered genera with the serum parameters in adult female offspring. **(A)** KEGG (Kyoto Encyclopedia of Genes and Genomes) database pathways were used to predict the microbial functional features at levels 2 and 3 among three groups in the adult female offspring. **(B)** Heatmap of Spearman correlation analysis between the significantly different germs at the genus level with serum biochemical parameters among different groups in the adult female offspring. N = 5/group. *p < 0.05 and **p < 0.01 indicate significant Spearman correlation. CD, control diet; HFD, high-fat diet; MS, milk fat globule membrane supplementation.

In the female offspring, the gut microbiota of the HFD group was characterized by low-representation of pathways involved in the digestive, endocrine, and excretory systems, transport and catabolism, metabolic diseases relative to the CD group. In contrast, neonatal MFGM supplementation shifted the profile of these functional pathways towards those observed in the CD group (**Figure 7A**). On the level 3 of KEGG, compared with the CD group, the low represented KEGG pathways in the HFD group were mainly in biological processes associated with lipid metabolism. These pathways included sphingolipid metabolism, linoleic acid metabolism, glycosphingolipid biosynthesis, and bile acid metabolism. However, the reduced richness of these pathways in the HFD group was increased in the HFD-MS group (**Figure 7A**). Notably, pathways involved in energy metabolism, such as glycolysis/gluconeogenesis, oxidative phosphorylation, citrate cycle, and electron transfer carries, have not been observed as high represented in female offspring as in male offspring.

### Correlation Between Gut Microbiota and Serum Biochemical Parameters in Adult Male and Female Offspring

To analyze the relationship between gut microbiota and biochemical metabolism in adult offspring, we performed a correlation analysis between the serum biochemical parameters and significantly different microbiota at the genus level. In the adult male offspring, TG and AST were found to be positively correlated with the relative abundance of *g-Bacteroides*, *g-Rikenella*, *g-Alloprevotella*, and *g-Muribaculum* (P < 0.05), but were negatively correlated with the relative abundance of *g-Parabacteroides*, *g-Bifidobacterium*, *g-Faecalibaculum*, and *g-Lactobacillus* (P < 0.05 or P < 0.001). TC and ALT were negatively correlated with the relative abundance of *g-Bifidobacterium*, *g-Faecalibaculum*, and *g-Prevotella* (P < 0.05 or P < 0.01). In contrast, the relative abundance of *g-Bifidobacterium* and *g-Faecalibaculum* was positively correlated with HDL-C in adult male offspring (P < 0.05) (**Figure 6B**). In the adult female offspring, AST was positively correlated with the abundance of *g-Rikenella* and *g-Ruminiclostridium* (P < 0.05 or P < 0.01). HDL-C was negatively correlated with *g-Rikenella* and *g-Ileibacterium* (P < 0.05) (**Figure 7B**). Collectively, we found that the characteristic bacteria in adult male offspring played a significant role in regulating lipid metabolism. However, the association between the characteristic bacteria and serum parameters did not appear to be prominent in the adult female offspring.

## Discussion

Gut microbiota, which is transmitted from mother to offspring, can affect the host’s energy homeostasis, nutrient acquisition, and metabolism. Thus, the influence of maternal HFD during gestation and lactation on the offspring gut microbiota may be an important factor regulating metabolism. Compelling evidence has demonstrated that gut microbiota dysbiosis plays an important role in linking adverse early life environments and metabolic disorders in later life (Codagnone et al., 2019). In accordance with a previous study (Zhou et al., 2019), the present study showed that maternal HFD during pre-pregnancy, pregnancy, and lactation results in significant glucose intolerance, insulin resistance, disorders of serum lipid profiles, and microbiota dysbiosis in adult mice offspring even when offspring are switched to a control diet after weaning.

MFGM contains large amounts of bioactive ingredients, such as unique polar lipids and membrane-specific proteins, and contributes to the growth and development of infants (Hernell et al., 2016). In a rodent model, the supplementation of polar lipid-enriched MFGM to obese dams was demonstrated to increase energy expenditure and alleviate obesity in adult offspring exposed to maternal HFD and an obesogenic environment post-weaning (Li et al., 2020). Although several groups have focused on the relationship between prenatal/postnatal MFGM supplementation and adult metabolic health, we are committed to identifying the mechanism behind it. In this study, we found that neonatal supplementation with MFGM during suckling improved glucose and lipid metabolism in both male and female adult offspring challenged with maternal HFD during pregnancy and lactation. Given the crucial role of gut microbiota in metabolic health, we performed 16s rRNA sequencing of fecal flora in adult male and female offspring and compared the microbiota among the groups. The results revealed that maternal HFD induced fecal microbiota dysbiosis in the HFD group, and that MFGM supplementation during suckling could partially restore the dysbiosis caused by maternal HFD. Of note, the adult offspring in our study demonstrated remarkable sexual dimorphism in metabolism and gut microbiota.

Sex is an important biological variable that can markedly impact multiple physiological and pathological processes (Arnold et al., 2017). Recently, more and more attention has been paid to explore differences based on sex, with male and female infants having been demonstrated to respond differently to the same environmental stimuli, with different growth and developmental trajectories in many cases (Cheng et al., 2016; Chowen et al., 2018). In terms of the metabolism, sex differences are usually observed in the form of greater insulin sensitivity, higher fatty acid oxidation, and lower serum TG levels in female mice compared to male mice (Yokomizo et al., 2014). Some clinical and experimental evidence has confirmed that male offspring are more likely to develop obesity and insulin resistance than female offspring in response to maternal overnutrition. Reliable evidence has demonstrated that during critical periods of development, changes in the sex steroid environment have long-term effects on body weight and metabolism in both male and female mice (Nohara et al., 2013; Carrillo et al., 2016). More specifically, estrogen has been reported to have a protective effect on the predisposition of offspring exposed to early life adverse environments to metabolic diseases. In the present study, we found that the levels of serum TG in the male offspring from dams fed a HFD were significantly higher than in those from dams fed a control diet, which was subsequently greatly ameliorated by MFGM supplementation. However, there was no significant difference in the TG levels among the three groups in the adult female offspring.

To determine the effects of maternal HFD and MFGM supplementation on glucose metabolism in offspring, we measured the FBG and insulin levels in male and female offspring at the eleventh week after birth. Exposure to maternal HFD was found to have little effect on the fasting serum insulin levels in the male and female offspring, but increased the FBG levels in the male offspring. Upon stress resulting from extra glucose, male but not female offspring showed impaired glucose tolerance, which indicated the impaired function of beta cells in adult male offspring exposed to maternal HFD. However, the insulin sensitivity, which was indicated by IPITT, was found to be normal in both male and female offspring exposed to maternal HFD. These evidences suggested that maternal HFD exposure exhibited more detrimental effects on the function of pancreatic beta cells, rather than on the insulin sensitivity in peripheral organs. For neonatal interventions preventing the outcomes of maternal overnutrition, MFGM showed beneficial effects on glucose metabolism in both male and female offspring, although maternal HFD exposure only exhibited mild but not significant impact on glucose metabolism in the female offspring. Thus, whether the long-term effect of MFGM on glucose metabolism is gender-dependent or not cannot be deduced from our results, but one may speculate that MFGM could have shown beneficial effects if the female mice had been exposed to a more extreme environment.

The gut microbiota represents the collection of microorganisms that live in the gastrointestinal tract. The gut microbiome plays a pivotal role in host metabolism and is depicted by global ecological parameters, including the richness, diversity, and evenness of its microbial communities. Several studies have found that the consumption of HFD directly decreases the richness and diversity of the gut microbiome (Jing et al., 2018). However, in the present study, no significant differences in the α-diversity indices were observed among the three groups in both male and female offspring. We speculated that maternal HFD exposure rather than direct HFD consumption could be the main reason of evenness of gut microbiome among groups. Moreover, offspring have been shifted to a control diet for 8 weeks after weaning, which could also lead to the recovery of reduced bacterial diversity. Although there was no difference in microbial diversity among groups in either male or female offspring, the relative abundance of specific bacteria varied greatly among the groups. In healthy adults, the intestinal microbiome characteristics of the bacterial phylum showed that *Firmicutes* and *Bacteroidetes* were the two most predominant bacterial phyla, while *Proteobacteria*, *Actinobacteria*, *Fusobacteria*, and *Verrucomicrobia* were less represented phyla (Quigley, 2013). Additionally, perturbations in the proportional composition of *Firmicutes* and *Bacteroidetes* may provide insights into host health status (Gómez-Zorita et al., 2019). *Bacteroidetes* are the most common Gram-negative bacteria in the human gastrointestinal tract, and are considered to play an important role in the metabolism of dietary fiber due to functional capability of degrading polysaccharides and regulating calorie absorption (Wexler, 2007). With regards to *Firmicutes*, most of the gut bacteria representing this phylum are Gram-positive and can produce multiple short-chain fatty acids, which promote the health of the gastrointestinal tract and provide resistance to infection (den Besten et al., 2013). HFD has been commonly reported to alter the composition of microbiota by increasing *Firmicutes* and decreasing *Bacteroidetes*, resulting in a significantly higher F/B ratio (De Filippo et al., 2010; Gómez-Zorita et al., 2019). However, several studies have been unable to confirm these observations, and even showed that the F/B ratio* *was reduced in obese rodents (Tung et al., 2016; Zhou et al., 2017). Our study showed a notable sexual dimorphism in the changes of F/B ratio in response to maternal HFD, which illustrated that the F/B ratio of the HFD group was increased relative to the CD group in the female offspring, but that the ratio relative to the CD group was decreased in the male offspring. In line with our results, the sex-dimorphic response of F/B ratio to HFD exposure has been reported in human studies as well, which demonstrated that the increment of body mass index (BMI) caused an induction of F/B ratio in women, but a reduction in men (Haro et al., 2016). Therefore, sex differences cannot be ignored in the analysis of gut microbiota. Lastly, in the present study MFGM was found to attenuate the perturbation of bacterial composition induced by maternal HFD in a sex-specific manner.

Multiple microbial taxa have been implicated in maintaining proper physiological function. Previous studies have shown that the distinctive benefit of *Bifidobacterium* is its ability to modulate host defense responses and provide protection against infectious diseases (Saulnier et al., 2009). *Faecalibacterium*, an anaerobic and important butyrate-producing bacterium in the human colon, plays a crucial role in producing energy for colonocytes, as well as anti-inflammatory metabolites for mucosal defense (Ferreira-Halder et al., 2017). In our study, the relative abundances of the genera *Bifidobacterium* and *Faecalibacterium* were found to be significantly decreased in the male offspring of the HFD group, and MFGM supplementation moderately increased their abundance. Evidence has demonstrated the probiotic effects of some genera, such as *Lactobacillus* and *Parabacteroides*, on body weight and metabolism (Crovesy et al., 2017; Brusaferro et al., 2018). Some strains of *Lactobacillus* can modulate gut microbiota, resulting in weight loss and metabolic homeostasis (Crovesy et al., 2017). Moreover, *Parabacteroides* plays a predominant role in preventing obesity and ameliorating metabolic disorders and systemic inflammation (Wu et al., 2019). Our results showed that MFGM, an important active ingredient in milk, greatly increased the abundance of *Parabacteroides* in the HFD-MS group in both male and female offspring. However, for modulating *Lactobacilli*, the MFGM intervention most likely worked better in increasing its abundance in males than in females. Overall, neonatal MFGM supplementation was found to increase the number of favorable species in the gut microbiome of adults, which may contribute to the improvement of lifetime metabolism.

By analyzing the correlation between the metabolic parameters and the featured genera, we found that the genera *Bifidobacterium* and *Faecalibacterium* were strongly associated with favorable changes in lipid metabolic indices in male offspring, whereas these associations were absent in female offspring. These correlations indicate that *Bifidobacterium* and *Faecalibacterium* contributed greatly to improvements in lipid metabolism in response to MFGM intervention in male offspring. However, this contribution was limited in the female offspring. Other genera, such as *Lactobacillus* and *Parabacteroides*, showed a weaker association with favorable changes in lipid metabolism in the male offspring. In contrast, some bacteria, such as *Rikenella*, *Bacteroides, Alloprevotella*, and *Muribaculum*, were found to be positively correlated with the serum TG and AST levels. The tendency of *Bacteroides* and *Alloprevotella* in response to maternal HFD in our study was inconsistent with that reported by Zheng et al. (2016), which was repressed in response to maternal HFD. The male offspring in their study did not exhibit the phenotypes of glucose tolerance after exposure to maternal HFD, indicating that the metabolic status in their offspring was not comparable to that in our offspring. This metabolic difference may explain the microbiome disparity linked to maternal HFD between these studies. In addition, no featured genus was associated with changes in glucose metabolic indicators, FBG and insulin, in either male or female mice. This result implies that gut microbiota contributed less to the changes in glucose metabolism caused by maternal HFD and MFGM intervention compared to changes in lipid metabolism. The sex differences in the correlation analysis also suggested that the gut microbiota in the female offspring contributed less to the changes in serum metabolic traits caused by maternal diet and neonatal intervention compared to that in male offspring.

According to the functional prediction of fecal microbiota in the context of KEGG, we found several common metabolic pathways associated with lipid metabolism and energy regulation in both male and female mice. However, the KEGG prediction in the male offspring exhibited several unique metabolic pathways, including oxidative phosphorylation, citrate cycle, electron transfer carries, and ubiquinone biosynthesis, which are involved in maintaining mitochondrial function. These results prompted us to consider that fetal sex may influence mitochondrial metabolism, thus contributing to the divergent metabolic programming of male and female offspring challenged with maternal HFD. Numerous studies have shown that male infants are more likely to be disadvantaged when subjected to an adverse environment, in which mitochondrial dysfunction has been suggested to play a crucial role. John et al. reported higher levels of protein expression of mitochondrial complexes in the hearts of females compared to males (John et al., 2018). They went on to show that male but not female mice developed cardiac dysfunction in a model of type 2 diabetes. Larsen et al. have also shown that the mitochondria of cardiomyocytes in male offspring were more susceptible to excess circulating maternal fuels than that in female offspring (Larsen et al., 2019). They even proved that exposure to maternal HFD or diabetic pregnancy could persistently impair the mitochondrial fusion and fission balance in the developing hearts of male offspring.

## Conclusions

In conclusion, the results presented in this study suggest that a transmissible and modifiable interaction exists between gut microbiome and metabolism of offspring exposed to maternal HFD. Neonatal MFGM supplementation was demonstrated to improve metabolism in adult offspring and modulate the structure of gut microbiome. Intriguingly, the metabolic outcomes and the alteration of gut microbiome in offspring show sex dimorphism. However, further study with a larger sample size will be needed to elucidate the precise mechanism through which MFGM improves metabolism and regulates microbiota composition, as well as to determine specific genera play roles in this interaction.

## Data Availability Statement

The datasets presented in this study can be found in online repositories. The names of the repository/repositories and accession number(s) can be found below: NCBI BioProject; PRJNA699440.

## Ethics Statement

The animal study was reviewed and approved by the institutional review board and the animal care and use committee of Shanghai Xinhua Hospital.

## Author Contributions

YD and LQ: designed the research. YD and LQ: provided essential reagents and materials. QZ, LY, and LQ: performed research and analyzed the data. FX and BC bred the mice. LY and LQ wrote the paper. YD and LQ had primary responsibility for final content. All authors contributed to the article and approved the submitted version.

## Funding

This work was supported by Shanghai Key Laboratory of Pediatric Gastroenterology and Nutrition (grant number 17DZ2272000); Shanghai Natural Science Foundation (grant numbers 19ZR1442000, 18ZR1431100); National Natural Science Foundation (grant number 81630039); Foundation of Science and Technology Commission of Shanghai Municipality (grant number 19495810500); Foundation of Shanghai Municipal Health Commission (grant numbers shslczdzk05702, 2019ZB0101).

## Conflict of Interest

The authors declare that the research was conducted in the absence of any commercial or financial relationships that could be construed as a potential conflict of interest.

## Supplementary Material

Supplementary Figure 1 Comparison of estimator indices of α-diversity among groups. **(A)** Indices of α-diversity of the adult male offspring. **(B)** Indices of α-diversity of the adult female offspring. Data are expressed as the mean ± SEM. N = 5/group. CD, control diet; HFD, high-fat diet; MS, milk fat globule membrane supplementation.
