# Supplementary figures and images for: Neonatal Milk Fat Globule Membrane Supplementation During Breastfeeding Ameliorates the Deleterious Effects of Maternal High-Fat Diet on Metabolism and Modulates Gut Microbiota in Adult Mice Offspring in a Sex-Specific Way
Source: Front Cell Infect Microbiol. 2021 Mar 19;11:621957. doi: 10.3389/fcimb.2021.621957 (PMC8017235; doi:10.3389/fcimb.2021.621957)

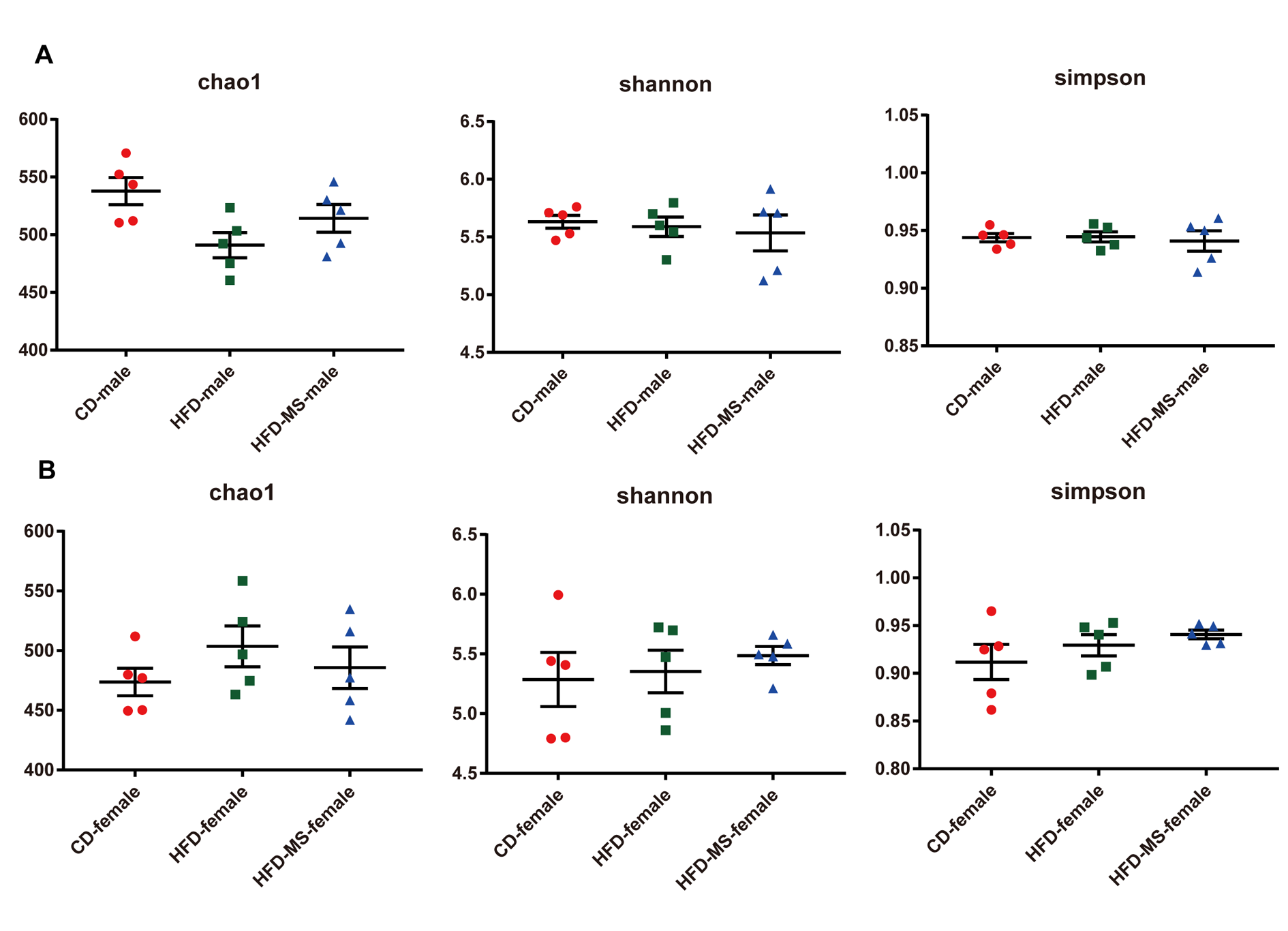

Supplement: Supplementary Figure 1 — Comparison of estimator indices of α-diversity among groups. (A) Indices of α-diversity of the adult male offspring. (B) Indices of α-diversity of the adult female offspring. Data are expressed as the mean ± SEM. N = 5/group. CD, control diet; HFD, high-fat diet; MS, milk fat globule membrane supplementation. [file Image_1.tif]
