# Supplementary material for: Neonatal Milk Fat Globule Membrane Supplementation During Breastfeeding Ameliorates the Deleterious Effects of Maternal High-Fat Diet on Metabolism and Modulates Gut Microbiota in Adult Mice Offspring in a Sex-Specific Way
Source: Front Cell Infect Microbiol. 2021 Mar 19;11:621957. doi: 10.3389/fcimb.2021.621957 (PMC8017235; doi:10.3389/fcimb.2021.621957)
Supplement: Supplementary file 2 [file Table_1.docx]

**Supplementary Table 1 | Diet composition**

| **Ingredients** |  | **Control diet** |  | **High fat diet** |
| --- | --- | --- | --- | --- |
|  |  | **(g/kg)** |  | **(g/kg)** |
| Corn Starch |  | 397.5 |  | 0 |
| Casein |  | 200 |  | 267 |
| Maltodextrin |  | 132 |  | 157 |
| Sucrose |  | 100 |  | 89 |
| Soybean Oil |  | 70 |  | 33 |
| Lard |  | 0 |  | 301 |
| Cellulose |  | 50 |  | 67 |
| Mineral Mix |  | 35 |  | 66 |
| Vitamin Mix |  | 10 |  | 13 |
| L-Cystine |  | 3 |  | 4 |
| Choline Bitartrate |  | 2.5 |  | 3 |
